# Insight into Fructose-to-Sucrose Ratio as the Potential Target of Urinalysis in Bladder Cancer

**DOI:** 10.3390/metabo14060345

**Published:** 2024-06-20

**Authors:** Dewang Zhou, Jianxu Huang, Haoxiang Zheng, Yujun Liu, Shimao Zhu, Yang Du

**Affiliations:** 1 Kobilka Institute of Innovative Drug Discovery, School of Medicine, The Chinese University of Hong Kong, Shenzhen 518172, China; dewangzhou@link.cuhk.edu.cn; 2 Shantou University Medical College, Shantou University, Shantou 515063, China; 22jxhuang@stu.edu.cn; 3 Department of Urology, Medical School, Shenzhen University, Shenzhen 518116, China; zhenghaoxiang2022@email.szu.edu.cn; 4 Medical School, Anhui University of Science and Technology, Huainan 232001, China; 2022201562@aust.edu.cn

**Keywords:** ATP-associated metabolites, bladder cancer, urinalysis, Mendelian randomization study

## Abstract

Bladder cancer usually has been diagnosed in elderly patients as it stays asymptomatic until it presents. Current detection methods for bladder cancer cannot be considered as an adequate screening strategy due to their high invasiveness and low sensitivity. However, there remains uncertainty about targets with high sensitivity and specificity for non-invasive bladder cancer examination. Our study aims to investigate the actionable non-invasive screening biomarkers in bladder cancer. Here, we employed scRNA-seq to explore the crucial biological processes for bladder cancer development. We then utilized bidirectional Mendelian randomization (MR) analysis to explore the bidirectional causal relationship between ATP-associated metabolites in urine and bladder cancer. Lastly, we used a BBN-induced mouse model of bladder cancer to validate the crucial gene identified by scRNA-seq and MR analysis. We found that (1) the ATP metabolism process plays a critical role in bladder cancer development; (2) there is a bidirectional and negative causal relationship between fructose-to-sucrose ratio in urine and the risk of bladder cancer; and (3) the higher expression of TPI1, a critical gene in the fructose metabolism pathway, was validated in BBN-induced bladder tumors. Our results reveal that fructose-to-sucrose ratio can serve as a potential target of urinalysis in bladder cancer.

## 1. Introduction

Bladder cancer is a highly heterogeneous disease with distinct pathology and molecular phenotypes [1,2,3]. Bladder cancer can be classified into two major types, non-muscle-invasive bladder cancer (NMIBC) and muscle-invasive bladder cancer (MIBC), based on tumor stage. Although most patients (70%) are initially diagnosed as NMIBC with a good prognosis, frequent recurrence and relapse progression largely limit our ability to completely cure NMIBC [4]. The patients with NMIBC need frequent evaluation with aggressive methods to diagnose relapse tumors [5]. The strict follow-up impairs their quality of life and increases their financial burdens. Patients with MIBC also typically accumulate the high costs produced by cystectomy and chemotherapy [4,5]. If bladder cancer can be detected with non-invasive techniques at an early or even pre-malignancy stage, overall survival and life quality of patients will be largely improved.

Currently, invasive cystoscopy is the major method for screening for bladder cancer [6], and this may cause urethral injury, urinary tract infection, and hematuria [7]. Clinicians also apply a non-invasive method (urine cytology) to detect cancer cells in urine, but it has a low sensitivity [8]. The advances in multi-omics have enabled the analysis of liquid biopsies through urinary metabolites, urinary free DNA, and urinary proteins [9]. Although genomics and proteomics have helped subtype many cancers and found many biomarkers, these biomarkers lacked good sensitivity and specificity because of considerable heterogeneity in tumor phenotypes and patient outcomes, even within the same genomic subtype, owing to unique cellular processes and metabolic profiles [10].

Emerging evidence has shown the changes in a class of urinary metabolites of patients with bladder cancer compared with healthy controls. However, some studies regarding changes in some metabolites are conflicting in patients with bladder cancer, as they have been described as both reduced [11,12] and increased [13]. These discrepancies might be owing to different measurement methods. Shao et al. [14] further emphasized the variability of putative biomarkers which are influenced by serval unmeasured confounders. Although researchers made efforts in statistics and methods to address them [15,16], these biases still persist and pose challenges to reliable designation of potentially effective and actionable screening targets.

Mendelian randomization (MR) estimates the potential causality between the exposure and the outcome based on associations between single-nucleotide polymorphisms (SNPs) and instrumental variables (IVs) [17]. Since genotypes are random during gamete formation, the involvement of the IV model largely resolves problems of confounding in observational studies, particularly the bias effects of unmeasured confounders on causal inference [18]. Therefore, MR enables effective identification of urinary biomarkers for screening of bladder cancer.

Here, we employed scRNA-seq and MR analysis to investigate potential urinary targets for non-invasive screening of bladder cancer, and further validated our approach in the N-butyl-N-(4-hydroxybutyl)-nitrosamine (BBN)-induced tumors of bladder cancer.

## 2. Materials and Methods

### 2.1. Study Design

**Figure 1:**
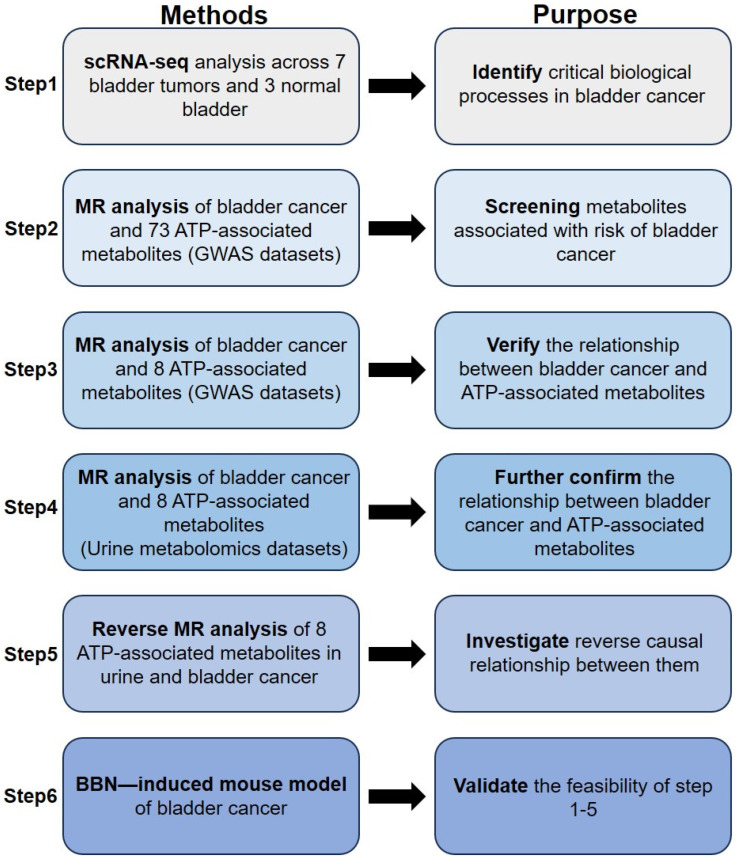
Study Design.

We initially leveraged single-cell RNA-seq (scRNA-seq) to identify the vital candidate biological processes in bladder cancer. Next, we employed two-sample Mendelian randomization to investigate the correlation between 73 ATP-associated metabolites from the genome-wide association study (GWAS) datasets and the risk of bladder cancer. After that, eight ATP-associated metabolites were identified. We reconducted the MR analysis to further explore the precise relationship between the eight ATP-associated metabolites and the risk of bladder cancer (negative correlation or positive correlation). We further utilized metabolism data to validate the above results from urine metabolomics datasets. Subsequently, to examine the possibility of reverse causality and mediating effects, we employed a reverse analysis of the impact of bladder cancer on eight ATP-associated metabolites using urine metabolomics datasets. Finally, we used a BBN-driven mouse model of bladder cancer to validate the feasibility of the above analyses. A schematic of the study is shown as a flowchart in Figure 1.

### 2.2. Single-Cell RNA-Seq Analysis

We downloaded the series GSE225190 dataset from GEO to obtain single-cell data from three samples of normal bladder tissue, and downloaded series GSE135337 to obtain single-cell data from seven samples of bladder cancer tissue.

The Seurat (v.4.3.0) workflow was applied for the analysis and visualization of single-cell RNA-seq data. Genes expressed in fewer than three cells were not considered, and cells expressing between 300 and 7000 genes with less than 10% mitochondrial proportion were retained for further analysis. All datasets were merged using the Seurat “merge” function, and principal component analysis was performed using 4000 highly variable genes identified with the “vst” mode.

### 2.3. Genetic Variation of Metabolites Related to ATP Metabolism

We obtained the exposures of 73 metabolites related to ATP metabolism from the EMBL-European Bioinformatics Institute. Further filter for significantly associated single nucleotide polymorphisms (SNPs (official name)) as instrumental variables not single nucleotide variants (SNVs), all meeting the genome-wide significance threshold (*p* < 1 × 10^−5^, linkage disequilibrium [LD] R2 > 0.001 within a 10,000 kb window). *p*-values were calculated by the Chi-square test. The F-statistics was computed to assess weak instrument bias and remove weak instrumental variables (F statistic > 10). Table S1 presents comprehensive information on the 73 ATP-associated metabolites.

### 2.4. GWAS Data for Outcome

The genome-wide association study (GWAS) datasets for bladder cancer of the European population were obtained from the IEU GWAS database (https://gwas.mrcieu.ac.uk/ (accessed on 5 January 2024)).

### 2.5. Acquisition of Urine Metabolomics Data

Urine metabolomics data consisting of 66 bladder cancer patients and 89 normal subjects were obtained from the literature to acquire differential metabolites between bladder cancer patients and normal cohorts [13]. Differential metabolites were screened with *p*-value < 0.05 and absolute logFC > 0. *p*-values were calculated by t-test and were FDR-corrected using the Benjamini–Hochberg procedure, and finally, associated metabolites were retained if they had an FDR below 0.1.

### 2.6. Immunohistochemistry and Histology

All paraffin-embedded tumor tissues were sectioned with 5 μm thickness. For histology, sections were dehydrated in Xylene (Aladdin, Shanghai, China, Cas# X139941) for 5 min thrice, 100% ethanol (Aladdin, Cas# E684328) for 3 min twice, 95% ethanol for 2 min, 70% ethanol for 2 min, and 50% ethanol for 2 min, and then were processed by Hematoxylin and Eosin (H&E) staining according to the standard protocol. For immunohistochemistry, the section of paraffin-embedded mouse bladder tissue was incubated in boiled sodium citrate buffer for 5 min in a high-pressure chamber (Biocare Medical, Pacheco, CA, USA). The tissues were then blocked with 10% goat serum for 30 min at room temperature, rinsed with PBST three times, and further incubated with the primary antibody (HUABIO, Guangzhou, China, Cat# HA500283, 1/400) for 60 min at room temperature. DAB (Thermo Scientific, Waltham, MA, USA, Cat# 34002) was used as the chromogen. Tissues were counterstained with hematoxylin and mounted with DPX media (Thermo Scientific, USA, Cat# X1525).

### 2.7. Statistical Analysis

The Mendelian randomization (MR) was conducted using the “TwoSampleMR” package (version 0.5.7) in R (version 4.2.2). Various MR approaches, including inverse variance weighted (IVW), weighted median, simple mode, weighted mode, and MR-Egger, were employed to investigate the potential causal effect of 73 ATP-associated metabolites on the risk of bladder cancer. The IVW method is the primary method. Only metabolites for which the OR values from all five methods are either all greater than 1 or all less than 1, and the *p* value for the IVW method is less than 0.05, are identified as metabolites associated with bladder cancer. If OR values are close to 1 in some studies of small sample size, the effects could be statistically significant when the *p* value is below 0.05. Therefore, we still use these data for further analysis. Additionally, if we use MR approaches to investigate the more complex questions like phenotypes, the OR values should be improved to increase reliability of results.

Instrumental variables from different analysis platforms, experiments, and populations may have heterogeneity, thus affecting Mendelian randomization analysis results. Heterogeneity is assessed by IVW and MR-Egger tests. Cochran Q-derived *p* < 0.05 indicates the presence of heterogeneity in the study. If the instrument variable affects the outcome through factors other than the exposure, then the instrument exhibits pleiotropy. Pleiotropy causes the independence and exclusion restriction assumptions to fail. The MR-Egger intercept test can detect pleiotropy in the data and assess the robustness of the results. If *p*-value < 0.05, it indicates that there is pleiotropy present in the data. The leave-one-out method was used to conduct a sensitivity analysis, with each SNP being removed in turn to observe whether the results changed after excluding each SNP to assess sensitivity.

### 2.8. Carcinogen-Induced Mouse Model of Bladder Cancer

In the experimental group, male C57BL/6J mice (Gempharmatech Co., Ltd., Nanjing, China) at eight weeks old received 0.05% (*w*/*v*) BBN in drinking water. In the control group, eight-weeks-old male C57BL/6J mice drank normal water without BBN. After four months, all bladders from two groups were collected, and then fixed in 10% formalin for 24 h for further H&E staining and immunohistochemistry.

## 3. Results

### 3.1. Single-Cell RNA-Seq Reveals the Critical Role of ATP-Associated Metabolism in Bladder Cancer Development

**Figure 2:**
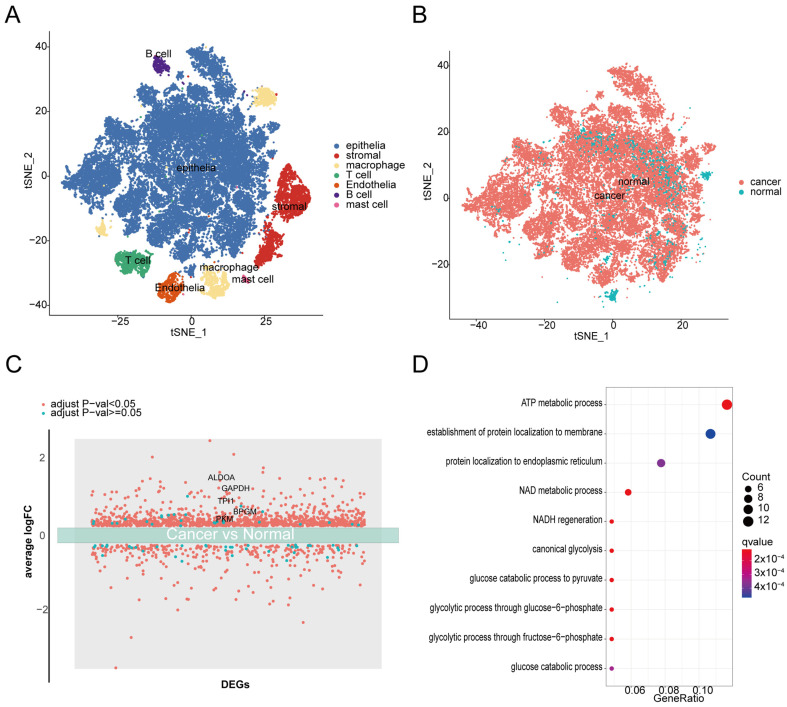
scRNA-seq analysis across bladder cancer samples and normal samples. (**A**) Gene expression tSNE and clustering of cells, colored by cell subtypes. (**B**) t-SNE map of the epithelial cells from tumor samples and normal samples. (**C**) Differentially expressed genes between epithelial cells from tumor samples and normal samples. (**D**) Gene ontology (GO) enrichment plot of the upregulated genes in tumor samples compared to normal samples.

To explore the effective screening targets in bladder cancer, we integrated the scRNA-seq data across seven bladder cancer samples and three normal samples, and then detected seven distinct subpopulations characterized by “SingleR” package and manual annotation: epithelial population, stromal population, macrophage population, T cell population, endothelial population, B cell population, and mast cell population (Figure 2A). Additionally, all epithelial cells in the seven bladder cancer samples were considered to be cancer cells based on inferred copy number variations (CNV) [19]. Therefore, we then re-subtyped the epithelial population into cancer cells and normal epithelial cells according to types of samples (Figure 2B), and further investigated differentially expressed genes (DEGs) between them and functions of DEGs through gene ontology (GO) enrichment (Figure 2C,D). GO enrichment indicated that the almost top 10 enriched biological pathways (BPs) are associated with cell metabolism (Figure 2D). From the enriched BPs, we found that ATP is involved in almost all activity of enriched BPs, and the ATP metabolism process has the highest enrichment level (Figure 2D). Collectively, ATP metabolism is a limiting process for cancer development and metabolic preferences of bladder cancer cells compared with normal cells.

### 3.2. Two-Sample Mendelian Randomization Analysis of 73 ATP-Associated Metabolites and Bladder Cancer

**Figure 3:**
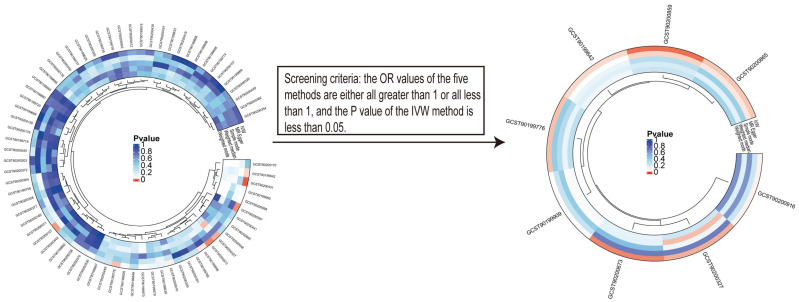
The process of screening metabolites related to bladder cancer. Circular heatmaps of ATP-associated metabolites display the Mendelian randomization results calculated by five methods (**left**) and filtered metabolites with a causal relationship (**right**).

Cells adapt ATP metabolism to support bladder tumor initiation and progression [20], but there remains uncertainty about which metabolites are associated with the risk of bladder cancer. To dissect ATP-associated metabolites related with the risk of bladder cancer, we leveraged a two-sample MR to investigate the causal impacts of 73 ATP associated metabolites on the risk of bladder cancer. Metabolites for which the OR values from all five methods (see statistical analyses in method) are either all more than 1 or all less than 1, and the *p* value on IVW method is less than 0.05, are identified as the metabolites associated with risk of bladder cancer. Finally, we obtained eight ATP-associated metabolites based on the filtering criteria shown in Figure 3. The detailed results of MR analysis are shown in Table 1.

### 3.3. Two-Sample Mendelian Randomization Analysis of Eight ATP-Associated Metabolites and Bladder Cancer

**Figure 4:**
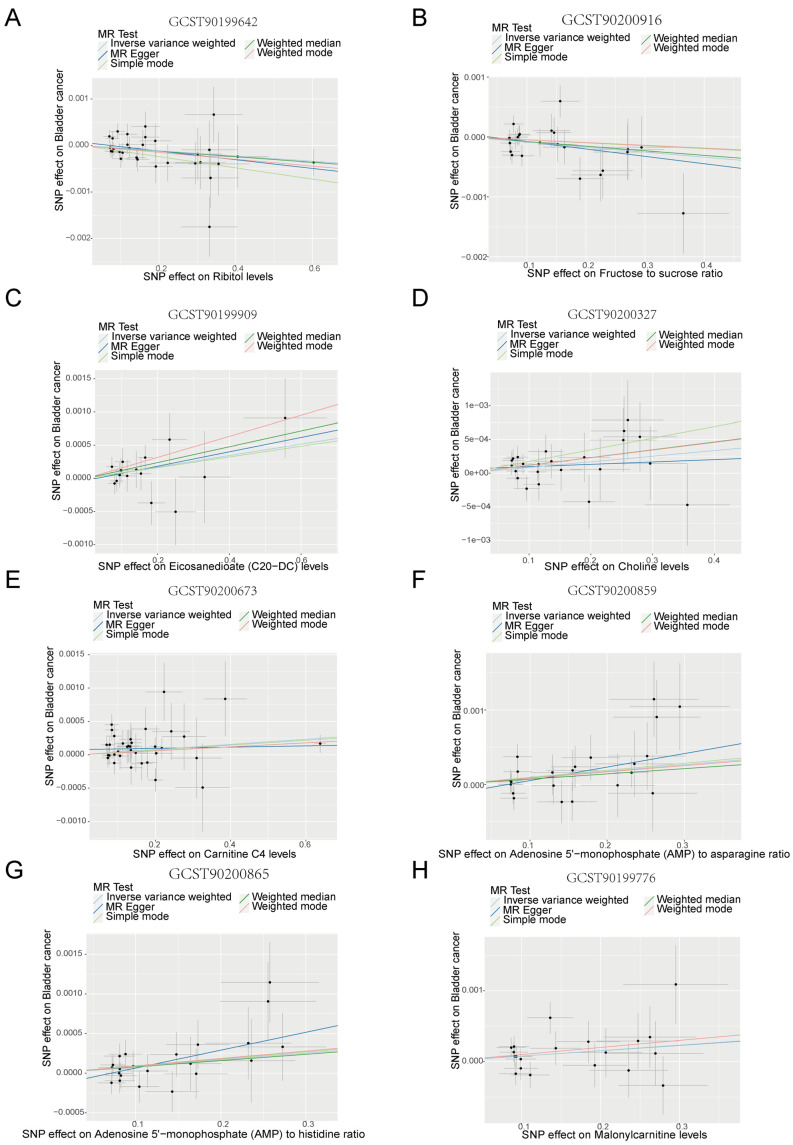
Two-sample MR analysis of eight ATP-associated metabolites and risk of bladder cancer. (**A**–**H**) Scatter plot for causal effects of eight ATP-associated metabolites on bladder cancer.

**Figure 5:**
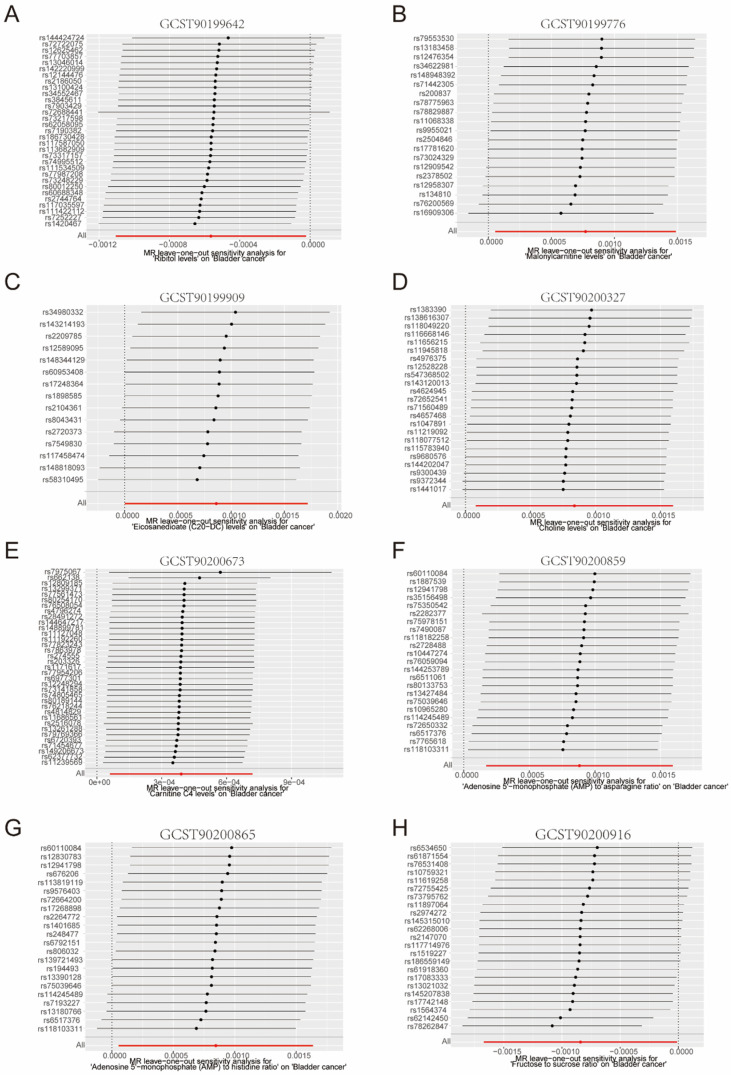
Sensitivity analysis of eight filtered ATP-associated metabolites. (**A**–**H**): Leave-one-out analysis on GCST90199642 (**A**), GCST90199776 (**B**), GCST90199909 (**C**), GCST90200327 (**D**), GCST90200673 (**E**), GCST90200859 (**F**), GCST90200865 (**G**), and GCST90200916 (**H**).

**Figure 6:**
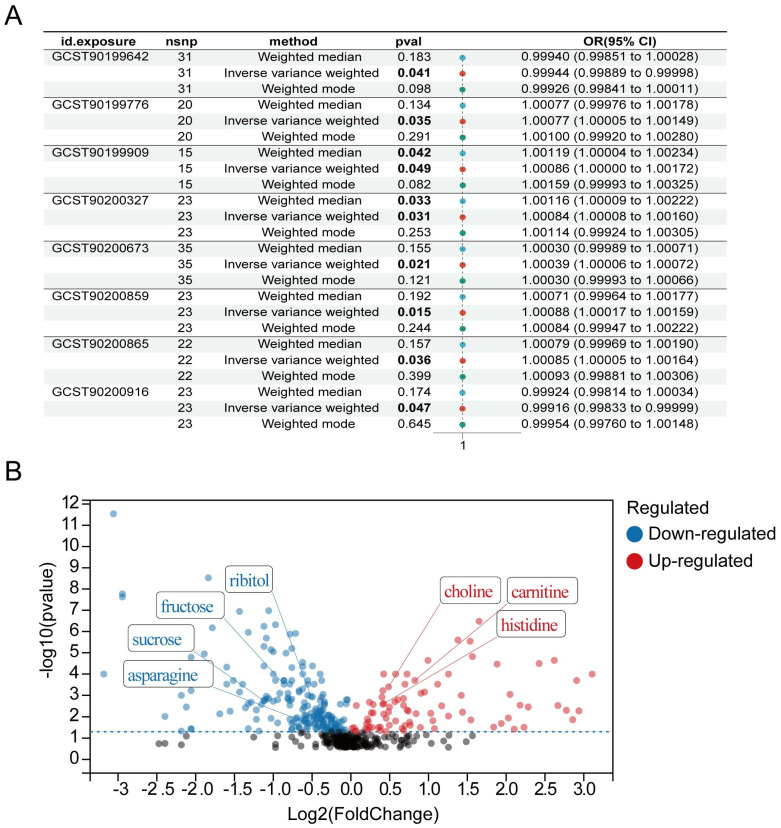
Two-sample MR analysis of eight ATP-associated metabolites in urine and bladder cancer. (**A**) Forest plots representing the MR estimates and 95% CI values of the causal effects of metabolites. (**B**) Volcano plot of differential metabolites.

To investigate the precise relationship between filtered metabolites and the risk of bladder cancer, we leveraged five MR methods to scrutinize the relationship between eight ATP-associated metabolites and the risk of bladder cancer. Our results revealed a negative correlation between GCST90199642 (Ribitol) and GCST90200916 (fructose-to-sucrose ratio) and the likelihood of bladder cancer (Figure 4A,B), and a positive correlation between the remaining six ATP metabolites and risk of bladder cancer (Figure 4C–H). Additionally, the leave-one-out analysis indicated that our results did not change significantly after excluding a single SNP, suggesting that the results were stable (Figure 5A–H). We further used three MR methods based on urine metabolomics data to investigate whether urinary metabolomics data matched previous results. Figure 6A shows that the results of MR analysis based on two different datasets are consistent. The volcano plot further revealed relative levels of specific urinary metabolites in patients with bladder cancer compared with healthy subjects (Figure 6B). Interestingly, levels of fructose and sucrose are both lower in bladder cancer patients, further requiring a combination of fructose and sucrose as a biomarker to elevate sensitivity and specificity for urinalysis.

### 3.4. Reverse Mendelian Randomization Analysis of Eight ATP-Associated Metabolites and Bladder Cancer

**Figure 7:**
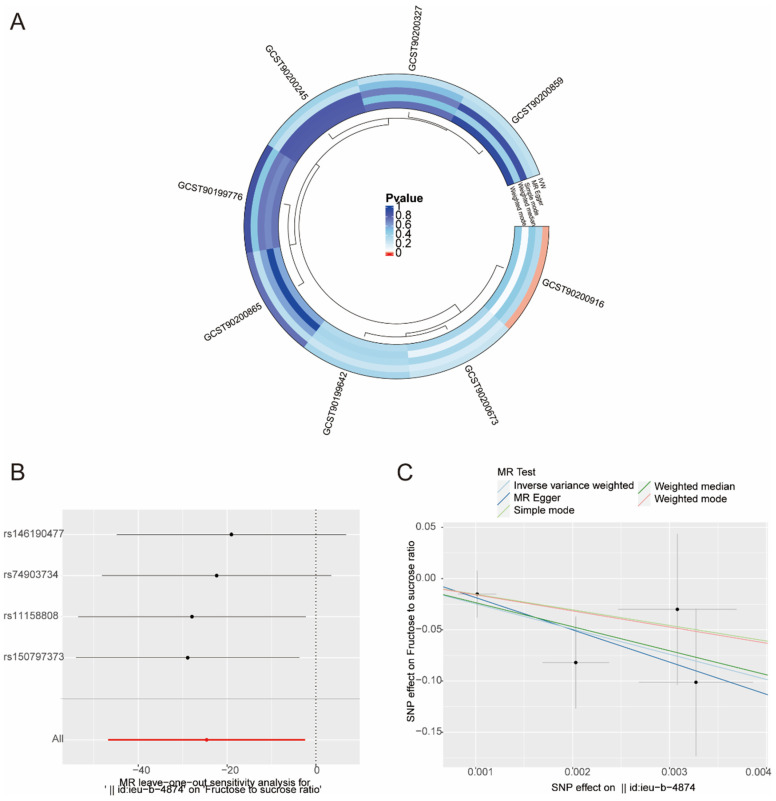
Reverse MR analysis of eight metabolites in urine and risk of bladder cancer. (**A**) Circular heatmaps of eight urinary metabolites calculated by MR. (**B**) Leave-one-out analysis. (**C**) Scatter plot for the causal effects of bladder cancer and fructose-to-sucrose ratio based on five MR methods.

To ascertain the potential reverse causal association between eight urinary metabolites and bladder cancer, a Mendelian randomization (MR) analysis was conducted with bladder cancer as the exposure variable and the eight urinary metabolites as the outcome variable. The results revealed a nonsignificant correlation between the two variables with *p* value greater than 0.05, except for GCST90200916 (fructose-to-sucrose ratio) (Figure 7A). The leave-one-out analysis demonstrated that the relationship between them was stable (Figure 7B). A scatter plot for the causal effects of bladder cancer level and fructose-to-sucrose ratio based on five MR methods revealed a strong negative relationship between them (Figure 7C).

### 3.5. Fructose-to-Sucrose Ratio Was Validated in a BBN-Induced Bladder Cancer Mouse Model

To further verify the feasibility and actionability of fructose-to-sucrose ratio as the urinalysis target, as mentioned previously, a volcano plot of DEGs associated with metabolism revealed that ALDOA, PKM, BPGM, GAPDH, and TPI1 were upregulated in bladder cancer (Figure 2C).

**Figure 8:**
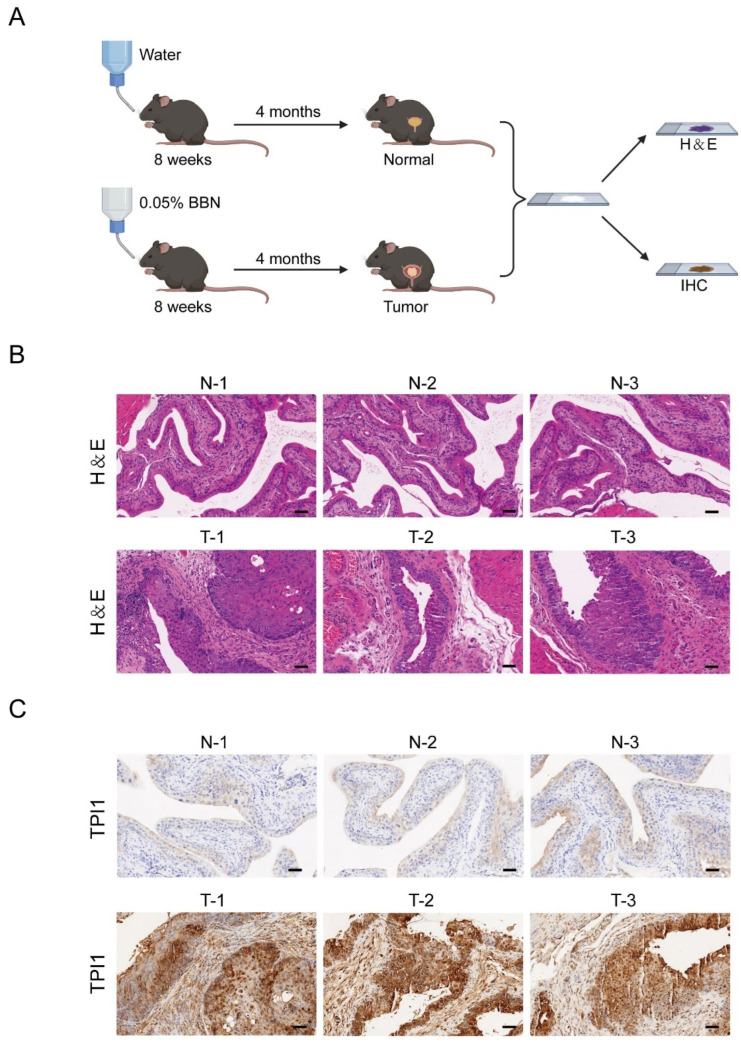
Expression of TPI1 was validated in BBN-induced mouse model of bladder cancer. (**A**) Schematic diagram of the generation of BBN-induced mouse model of bladder cancer and histological analyses. (**B**) Bladders from BBN and control group were collected and subjected to H&E staining. Normal: n = 3; tumor: n = 3. Scale bars, 50 μm. (**C**) TPI1 staining of bladder tissues from BBN and control group. Normal: n = 3; tumor: n = 3. Scale bars, 50 mm.

Among the above DEGs, only TPI1 and ALDOA are directly involved in the fructose metabolism pathway [21,22]. A study also indicated that ALDOA was upregulated in bladder cancer and promoted bladder cancer malignant progression, while there is no report associated with TPI1 in bladder cancer [23]. Therefore, we established a BBN-induced bladder cancer mouse model to detect expression of TPI1 in the tumors and the normal bladder tissues. As shown in Figure 8A, we studied mice either with or without 0.05% BBN, and then collected bladders from the control group and BBN group after four months. Next, the bladder tumors and normal bladder tissues were subjected to H&E staining and IHC. H&E staining and IHC results showed that TPI1 was upregulated in tumor tissues (Figure 8B,C). Collectively, these results indicated that fructose-to-sucrose ratio may be an ideal candidate screening target.

## 4. Discussion

In this study, we leveraged scRNA-seq across seven bladder tumors and three normal bladders to determine the critical role of the ATP metabolism process in bladder cancer development. Through forward MR study on 73 ATP-associated metabolites and the risk of bladder cancer, we did find that 2 ATP-associated metabolites (GCST90199642: Ribitol and GCST90200916: fructose-to-sucrose ratio) were negatively associated with risk of bladder cancer, and 6 ATP metabolites (GCST90199776: Malonylcarnitine; GCST90199909: Eicosenedioate (C20:1-DC); GCST90200327 Choline; GCST90200673: Carnitine C4; GCST90200859: Adenosine 5′-monophosphate (AMP)-to-asparagine ratio; GCST90200865: Adenosine 5′-monophosphate (AMP)-to-histidine ratio) were positively associated with risk of bladder cancer. The MR analysis of the above eight metabolites using urinary metabolomics data further supported their causal relationship. The reverse MR analysis further revealed a negative association between GCST90200916 (fructose-to-sucrose ratio) in urine and bladder cancer. BBN-induced bladder tumors confirmed higher expression of TPI1, a critical gene in the fructose metabolism pathway.

The advances in urine-based genomic and proteomic analysis enable the non-invasive generation of cellular morphology and both genomic and non-genetic information [24,25,26,27]. However, despite substantial progress in diagnosis marker research, the role of genomic and proteomic cancer biomarkers in clinical practice is still limited by the lack of either specificity or sensitivity [8,28]. Progress toward answering how to elevate the sensitivity and specificity of methods requires some new insights into cancer biology and urinalysis techniques. Following this notion, we applied scRNA-seq to investigate which biological processes are vital for bladder cancer development. GO enrichment analysis revealed the ATP metabolism process at the single-cell level was the most enriched in bladder cancer (Figure 2D). These results indicated that ATP-associated metabolites may be the potential candidate bladder cancer biomarkers. Indeed, metabolomics focuses on the more downstream products compared to genomic and proteomic, and thus most closely reflects a system’s phenotype [29,30]. Four MS studies on metabolic profiles from patients with bladder cancer and healthy subjects [11,12,31,32] showed excellent sensitivities and specificities (both 100%). Several studies also reported consistent results for the same metabolite in patients of bladder cancer using different combinations of metabolomics methods [11,13,31], indicating that targets of urinalysis based on metabolites are particularly convincing in terms of the stability of results.

Exploiting cell metabolism for clinical benefit requires understanding the contextual specificity of metabolic preferences. Some evidence showed fructose in urine from patients with bladder cancer at lower levels compared with healthy subjects [12,31]. A urine metabolomics study of rat bladder cancer showed an association between sucrose level and the risk of bladder cancer [33]. In this study, two-sample MR analysis revealed that fructose-to-sucrose ratio was negatively associated with risk of bladder cancer. If there is a bidirectional causal relationship between bladder cancer and metabolites, the metabolites will demonstrate more sensitivity and specificity as urinalysis biomarkers. To ascertain whether a reverse causality exists between fructose-to-sucrose ratio and bladder cancer, a reverse two-sample MR analysis was conducted with bladder cancer serving as the exposure factor and fructose-to-sucrose ratio in urine as the outcome. The findings indicated that there is a reverse causal relationship between them (Figure 7A–C). When fructose-to-sucrose ratio decreases, meaning the relative level of fructose decreases or the relative level of sucrose increases, risk of bladder cancer increases. Using a combination of fructose and sucrose level may be a feasible strategy for urinalysis of bladder cancer, but we need to determine the baseline of fructose-to-sucrose ratio as a reference.

As previously mentioned, urine metabolomics showed different results based on different platforms [11,12,31]. The greatest advantage in this study is that the causal estimate obtained by MR avoids confounding bias and elevates sensitivity by reverse causality analysis. Additionally, increasing the number of GWAS data in MR analyses can improve accuracy. In our study, we also used urinary metabolomics data from other platforms to successfully validate the results of MR analysis based on GWAS data (Figure 6A,B). However, our study still has several limitations. First, the results could not be generalized to other ethnicities and races because the population in our study was entirely European. Secondly, the number of IVs used in reverse MR is limited. We should conduct reverse MR analysis using a larger sample size to increase the reliability of data in the future. Finally, the sensitivity and specificity should be validated in a large bladder cancer community.

In conclusion, long-term exposure to some ATP-associated metabolites, such as malonylcarnitine and choline, may increase the risk of bladder cancer, but this requires further support from additional evidence in the future. Our bidirectional MR study showed a negative association between fructose-to-sucrose ratio in urine and the risk of bladder cancer, shedding light on a potential actionable target of urine screening. Given the fact that fructose and sucrose have different levels between patients with bladder cancer and healthy subjects, further investigations were employed in BBN-induced bladder tumors and normal bladders to confirm a higher expression of TPI1, a critical gene in the fructose metabolism pathway.

## Figures and Tables

**Table 1:** MR analysis for eight ATP-associated metabolites’ associations with bladder cancer risk.

ID	Exposure	Method	No. of SNPs	OR	95% CI	*p* Value
GCST90199642	Ribitol levels	MR-Egger	31	0.999064	0.99816~0.99997	0.052
		Weighted median	31	0.999396	0.99854~1.00025	0.167
		Inverse variance weighted	31	0.999435	0.99889~0.99998	0.041
		Simple mode	31	0.998796	0.9973~1.00029	0.125
		Weighted mode	31	0.999261	0.9984~1.00012	0.103
GCST90200673	Carnitine C4 levels	MR-Egger	35	1.000092	0.9996~1.00058	0.716
		Weighted median	35	1.000298	0.9999~1.0007	0.147
		Inverse variance weighted	35	1.00039	1.00006~1.00072	0.021
		Simple mode	35	1.000362	0.99928~1.00145	0.517
		Weighted mode	35	1.000297	0.99991~1.00069	0.142
GCST90199776	Malonylcarnitine levels	MR-Egger	20	1.000758	0.99885~1.00267	0.446
		Weighted median	20	1.000772	0.99976~1.00179	0.135
		Inverse variance weighted	20	1.000771	1.00005~1.00149	0.035
		Simple mode	20	1.000998	0.99908~1.00292	0.321
		Weighted mode	20	1.000998	0.99918~1.00282	0.295
GCST90200327	Choline levels	MR-Egger	23	1.000352	0.99851~1.0022	0.713
		Weighted median	23	1.001155	1.00009~1.00222	0.034
		Inverse variance weighted	23	1.000838	1.00008~1.0016	0.031
		Simple mode	23	1.00173	0.99969~1.00378	0.111
		Weighted mode	23	1.001141	0.99932~1.00296	0.232
GCST90200865	Adenosine 5′-monophosphate (AMP) to histidine ratio	MR-Egger	22	1.002257	1.0002~1.00432	0.044
		Weighted median	22	1.000794	0.99968~1.00191	0.161
		Inverse variance weighted	22	1.000846	1.00005~1.00164	0.036
		Simple mode	22	1.000893	0.99876~1.00303	0.421
		Weighted mode	22	1.000931	0.99887~1.003	0.386
GCST90200859	Adenosine 5′-monophosphate (AMP) to asparagine ratio	MR-Egger	23	1.001818	1.0002~1.00344	0.039
		Weighted median	23	1.000706	0.99961~1.0018	0.205
		Inverse variance weighted	23	1.000882	1.00017~1.00159	0.015
		Simple mode	23	1.000947	0.99916~1.00274	0.312
		Weighted mode	23	1.000841	0.99944~1.00225	0.254
GCST90200245	Eicosenedioate (C20:1-DC) levels	MR-Egger	19	1.000108	0.99889~1.00133	0.865
		Weighted median	19	1.000867	0.99993~1.00181	0.071
		Inverse variance weighted	19	1.000743	1.00007~1.00141	0.03
		Simple mode	19	1.000731	0.99887~1.0026	0.452
		Weighted mode	19	1.000931	0.99998~1.00188	0.07
GCST90200916	Fructose to sucrose ratio	MR-Egger	23	0.998772	0.99675~1.0008	0.248
		Weighted median	23	0.999235	0.99812~1.00035	0.18
		Inverse variance weighted	23	0.99916	0.99833~0.99999	0.047
		Simple mode	23	0.999513	0.99748~1.00155	0.644

## Data Availability

It is worth noting that the data analyzed in this study are publicly available and can be accessed via the IEU GWAS database (https://gwas.mrcieu.ac.uk/ (accessed on 5 January 2024)) and urine metabolomics database [13]. Figure 8A was created with BioRender.com (agreement number: SU26MFWRIS).

## Supplementary Materials

The following supporting information can be downloaded at: https://www.mdpi.com/article/10.3390/metabo14060345/s1, Table S1: Summary of 73 ATP associated metabolites datasets.
