# Supplementary material for: Insight into Fructose-to-Sucrose Ratio as the Potential Target of Urinalysis in Bladder Cancer
Source: Metabolites. 2024 Jun 20;14(6):345. doi: 10.3390/metabo14060345 (PMC11205578; doi:10.3390/metabo14060345)
Supplement: Supplementary file 1 [file metabolites-14-00345-s001.zip › metabolites-2955972-supplementary.pdf]

Table S1. Summary of 73 ATP associated metabolites datasets.

Exposure	ID	Population	Sample size
Glycerophosphorylcholine (GPC) levels	GCST90199629	European	8212
Glycerol 3-phosphate levels	GCST90199638	European	8201
Ribitol levels	GCST90199642	European	8128
Palmitoylcarnitine levels (Metabolon platform)	GCST90199666	European	8256
Erythritol levels in elite athletes	GCST90199667	European	8167
Hexanoylcarnitine levels (Biocrates platform)	GCST90199668	European	8242
Acetylcarnitine levels (Biocrates platform)	GCST90199669	European	8262
Isobutyrylcarnitine (c4) levels	GCST90199698	European	8243
Tauro-beta-muricholate levels	GCST90199719	European	4661
Octanoylcarnitine (c8) levels	GCST90199720	European	8226
Carnitine C14 levels	GCST90199730	European	8241
N-acetylhistidine levels	GCST90199735	European	7054
N-acetylproline levels	GCST90199737	European	7100
N4-acetylcytidine levels	GCST90199758	European	7425
Oleoylcarnitine levels	GCST90199774	European	8260
Malonylcarnitine levels	GCST90199776	European	5822
1-palmitoyl-GPE (16:0) levels	GCST90199792	European	8276
Mannitol/sorbitol levels	GCST90199818	European	8211
Hexanoylglutamine levels	GCST90199833	European	8128
Succinylcarnitine levels	GCST90199848	European	8212
21-hydroxypregnenolone disulfate levels	GCST90199849	European	8132
N-acetyl-3-methylhistidine levels	GCST90199868	European	7018

Sphingomyelin (d18:1/18:1, d18:2/18:0) levels	GCST90199878	European	8267
Cis-4-decenoylcarnitine (C10:1) levels	GCST90199880	European	8239
2s,3R-dihydroxybutyrate levels	GCST90199882	European	8252
Margaroylcarnitine (C17) levels	GCST90199929	European	8112
Octadecenedioylcarnitine (C18:1-DC) levels	GCST90199970	European	8245
Octadecanedioylcarnitine (C18-DC) levels	GCST90199992	European	8049
Sphingomyelin (d18:1/20:2, d18:2/20:1, d16:1/22:2) levels	GCST90199995	European	8124
Sphingomyelin (d18:1/20:1, d18:2/20:0) levels	GCST90199996	European	8255
Palmitoylcholine levels	GCST90200071	European	8209
Ceramide (d18:1/24:1) levels	GCST90200098	European	8265
Gamma-glutamyl-alpha-lysine levels	GCST90200107	European	8250
Behenoylcarnitine (C22) levels	GCST90200127	European	7368
Linoleoylcholine levels	GCST90200130	European	8193
Arachidonoylcarnitine (C20:4) levels	GCST90200150	European	8202
Sphingomyelin (d17:1/14:0, d16:1/15:0) levels	GCST90200169	European	8267
Octadecadienedioate (C18:2-DC) levels	GCST90200170	European	8231
Glyco-beta-muricholate levels	GCST90200179	European	7206
Indoleacetoylcarnitine levels	GCST90200203	European	7777
Undecenoylcarnitine (C11:1) levels	GCST90200236	European	8253
Dihomo-linoleate (20:2n6) levels	GCST90200320	European	8284
Glycerol levels	GCST90200325	European	8285
Choline levels	GCST90200327	European	8262
3-phosphoglycerate levels	GCST90200329	European	8196
1-palmitoyl-2-linoleoyl-gpc (16:0/18:2) levels	GCST90200330	European	8230

Laurate (12:0) levels	GCST90200350	European	8226
Linoleate (18:2n6) levels	GCST90200354	European	8260
Arachidonate (20:4n6) levels	GCST90200358	European	8272
Succinate levels	GCST90200362	European	7943
Cysteinylglycine levels	GCST90200364	European	8214
Aspartate levels	GCST90200370	European	8253
Arginine levels	GCST90200372	European	8237
Histidine levels	GCST90200377	European	8223
Methionine levels	GCST90200391	European	8222
Palmitate (16:0) levels	GCST90200397	European	8269
Glutamate levels	GCST90200412	European	8287
Glutamine levels	GCST90200419	European	8253
Cytidine levels	GCST90200429	European	7659
Alanine levels	GCST90200431	European	8265
Cysteine levels	GCST90200439	European	8216
Tryptophan levels	GCST90200441	European	8235
Valine levels	GCST90200442	European	8247
N-stearoyl-sphinganine (d18:0/18:0) levels	GCST90200448	European	6737
Sucrose levels	GCST90200449	European	7803
Pentadecanoate (15:0) levels	GCST90200457	European	8273
Carnitine C4 levels	GCST90200673	European	8111
1-stearoyl-2-arachidonoyl-gpc (18:0/20:4) levels	GCST90200685	European	8253
Glycine levels	GCST90200707	European	8262
Adenosine 5'-monophosphate (AMP) to asparagine ratio	GCST90200859	European	8188

Adenosine 5'-monophosphate (AMP) to histidine ratio	GCST90200865	European	8180
Phosphate to glycerol ratio	GCST90200903	European	8179
